# The Mouse Age Phenome Knowledgebase and Disease-Specific Inter-Species Age Mapping

**DOI:** 10.1371/journal.pone.0081114

**Published:** 2013-12-03

**Authors:** Nophar Geifman, Eitan Rubin

**Affiliations:** Department of Microbiology, Immunology and Genetics, Faculty of Medical Sciences and The National Institute of Biotechnology in the Negev, Ben Gurion University, Beer-Sheva, Israel; University of Illinois-Chicago, United States of America

## Abstract

**Background:**

Similarities between mice and humans lead to generation of many mouse models of human disease. However, differences between the species often result in mice being unreliable as preclinical models for human disease. One difference that might play a role in lowering the predictivity of mice models to human diseases is age. Despite the important role age plays in medicine, it is too often considered only casually when considering mouse models.

**Methods:**

We developed the mouse-Age Phenotype Knowledgebase, which holds knowledge about age-related phenotypic patterns in mice. The knowledgebase was extensively populated with literature-derived data using text mining techniques. We then mapped between ages in humans and mice by comparing the age distribution pattern for 887 diseases in both species.

**Results:**

The knowledgebase was populated with over 9800 instances generated by a text-mining pipeline. The quality of the data was manually evaluated, and was found to be of high accuracy (estimated precision >86%). Furthermore, grouping together diseases that share similar age patterns in mice resulted in clusters that mirror actual biomedical knowledge. Using these data, we matched age distribution patterns in mice and in humans, allowing for age differences by shifting either of the patterns. High correlation (r^2^>0.5) was found for 223 diseases. The results clearly indicate a difference in the age mapping between different diseases: age 30 years in human is mapped to 120 days in mice for Leukemia, but to 295 days for Anemia. Based on these results we generated a mice-to-human age map which is publicly available.

**Conclusions:**

We present here the development of the mouse-APK, its population with literature-derived data and its use to map ages in mice and human for 223 diseases. These results present a further step made to bridging the gap between humans and mice in biomedical research.

## Introduction

The mouse is intensively used as a model organism for the study of human disease [1]; Mice and humans share a strong similarity both physiologically and genetically, leading to the generation of many mouse models of human disease [2]. These are used to investigate disease mechanisms or to test the effect of intervention. Mice are often used in preclinical trials and most clinical trials are only initiated after an intervention was found to benefit mice models of a human disease.

While a marked likeness does exist between humans and mice, there are many significant differences. For example, differences exist between mice and humans in immune system development, activation, and response to challenge [3]. Such differences may also be expressed in the manifestation of disease such as multiple sclerosis which likely involves an autoimmune component [4].

The differences between mice and humans result in mice being too often unreliable as preclinical models for human disease; there are many examples of therapies that were found to be effective in a mouse model but failed in human clinical trials. One such example is the use of vaccination for the amyloid beta peptide, which was found to safely lead to clearance of amyloid plaques in an Alzheimer's Disease (AD) mouse model. In humans, the same treatment was found to cause severe and potentially lethal side effects, such as microhemorrhage and meningoencephalopathy [5].

One difference that might play a role in lowering the predictivity of mice models to human diseases is age. Despite the important role age plays in medicine, it is too often considered only casually when considering mouse models. Age is an important factor when considering phenotypic changes in health and disease; a patient's age can affect the course and progression of a disease [6], [7] or can be important in determining the correct course of treatment [8]. As with humans, the age of the mice may play a critical role in phenotypic manifestations and response to treatment. For example, in several mouse models for AD, the formation of amyloid β plaques depends highly on the age of the examined mouse. In the mutant β-amyloid precursor protein (APP), known as the London model for AD, plaques are usually observed around the age of 12–15 months while in the APP/London X Presenilin-1 double-transgenic mice the same plaques are observed at the age of 6 months [9].

The optimal age at which mice are investigated must take into account considerations such as the burden of keeping the mice until they age. However, due to the many innate differences between mice and humans, correctly choosing the appropriate ages in mice that would correspond to those in humans in the context of a specific disease is not trivial. Good age mapping can thus alleviate some of the differences between mice and men, and improve, if only a little, the predictive power of such models. In one example, mapping of embryonic ages between humans and murine models was suggested to be important for the applicability of the conclusions of radiation studies to humans [10]. We propose that fewer findings might be lost in translation if more careful choice of mouse age ranges will be used, specifically optimizing the age ranges for each disease model.

Recently, we developed the Age-Phenome Knowledge base (APK) that holds a structured representation of knowledge derived from the scientific literature and clinical data regarding clinically-relevant traits that occur at different ages [11]. The database underpinning the APK contains over 35,000 entries that describe relationships between age and disease, which were mined from over 1.5 million PubMed abstracts [12]. Using several analytic techniques, data stored in the Age-Phenome Knowledge base was used to examine age-disease relationships. In previous work we demonstrated that meaningful age groups can be redefined based on data derived from the bio-medical literature. These new age ranges are potentially better suited to describe important ages in the context of patient health. We further showed that the age groups are context-specific and differ between disease types. Furthermore, we showed that by grouping diseases together based on their occurrence in age, new hypotheses regarding links between diseases can be generated [13].

Here we describe the mouse-APK, an extension of the APK that stores knowledge about age-related phenotypic patterns in mice. The knowledgebase holds a structured representation of literature-derived knowledge about human disease and phenotypes occurring and researched at different ages in mice. Similarly to the APK, in the mouse-APK diseases and phenotypes are described using ontologies and standard vocabularies. The knowledgebase was populated, using computational text-mining tools, with data derived from over half a million mouse-related PubMed abstracts. Furthermore, a simple user-friendly query interface is also provided.

We present here the development of the mouse-APK, its population with literature-derived data, the analysis of age-related disease patterns in mice and their comparison to similar patterns in humans. By capturing age-disease relationships in mice as they are represented in the research corpus, a further step is made to bridging the gap between humans and mice in biomedical research.

## Methods

### Database

The database developed was conducted in MySQL [14]. Similarly to the APK [11], the mouse-APK database comprises of three main tables: (i) an *evidence* table that contains evidence instances (e.g. text fragments from abstracts) and their description; (ii) an *evidence-age* table that contains a description of the age information found in each evidence instance; and (iii) an *evidence-phenotype* table that links phenotypes to each evidence instance.

### Knowledgebase interfaces

The mouse-APK data browser was developed in HTML and PHP and can be found at: http://rubinlab.bgu.ac.il/mouseapk/mouseDataBrowser/.

The mouse-to-human age-map was developed in HTML and PHP and can be found at: http://rubinlab.bgu.ac.il/mouseAPK/H2M/.

### Text mining

The same text mining pipeline employed in [12] was used here, with minor alterations. Briefly, PubMed [15] abstracts were exported from the NCBI (April 2012) database, using the key word ‘mouse’. Regular expressions were used to find abstracts that mention a specific age or age range and retrieve the mentioned age or age range, using a one day resolution to represent age (and not 1 month as they were captured in APK). Text snippets were then generated from each abstract (usually 1–3 sentences) that best captured the most relevant information for each abstract. Text snippets were mapped to disease names and their synonyms as defined in the Disease Ontology (DO) [16], as well as to a selected list of concept names and synonyms from the Unified Medical Language System (UMLS) [17]. In addition, various mouse strains are also sought after and gender is assigned to an evidence instance if only one gender is mentioned in the text snippet. The scripts used for this purpose and all the necessary accessory files, are available as File S1, File S2, File S3 and File S4.

### Text mining data quality evaluation

Evaluation of the quality of the data captured from PubMed abstracts and stored in the databases was performed by comparing the results of the knowledge-mining processes to those achieved by a human reader.

250 randomly selected abstracts were used to evaluate the task of finding age-related abstracts. All abstracts were read by a human evaluator who decided whether an abstract included age-related information. Abstracts were considered to be age-related only if a specific age or age range was mentioned (e.g. age 3 months).

To evaluate the performance of the mining pipeline in extracting a textual snippet from an abstract and mapping to diseases and additional phenotypes, 137 instances that were linked to Alzheimer's disease were reviewed manually. The results of the human reader in all mining tasks were then compared to the results obtained by the computational mining pipeline and measured in terms sensitivity (recall) and specificity (File S5).

### Mapping ages by pattern correlation

A quantitative age-disease matrix was generated for the APK by obtaining, for each disease mapped in the database, a count of the number of instances mapped to that disease per age. Evidence linked to inferred age ranges (e.g. “under age 30” or “over age 22”) were excluded from this analysis. A similar matrix was generated using the mouse-APK data, albeit with a one day age resolution (File S6). Each of the matrices were normalised by dividing the cells for each disease by the disease total instances count to control for disease over- or under-representation in the literature.

**Figure 1:**
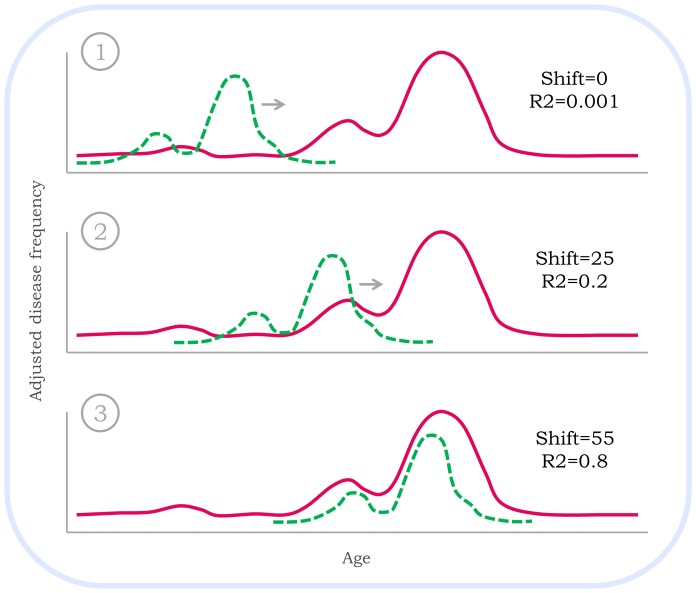
Schematic illustration of the process for the calculation of correlation. A correlation between the human and mouse disease-age pattern is calculated allowing for the human pattern (green dashed line) to be shifted against the mouse pattern (pink solid line). The shift which yields the best correlation is selected as the best shift (3).

For each disease for which data was available from both matrices (human and mouse) a correlation was sought between the two data vectors (Figure 1). A script implemented in R calculated the Pearson correlation between each set of patterns (for each disease). Correlations were calculated for shifted patterns (vectors), allowing for the human vector to be shifted against the mouse vector for its entire length (up to a 1028 day shift).

To select the correct resolution for age in mice, a correlation between disease patterns in mice and matching pattern in humans was calculated for both one-day resolution and one-week resolution in mice. The one-day resolution for mouse ages yielded a higher number of correlating disease patterns with the human-derived data and was thus selected for the analysis. The full details of the comparison are provided in File S7.

### Hierarchal cluster analysis

A quantitative disease-age matrix was generated as described above. Using Expander 5 software [18], hierarchal clustering was conducted (with default parameters, using Pearson correlation as the similarity statistic). Only diseases which have at least five database instances were included in the analysis.

### Availability

All the material pertaining to this project is freely available at:

http://rubinlab.bgu.ac.il/mouseAPK.

The mouse-APK data browser can be found at: 

http://rubinlab.bgu.ac.il/mouseapk/mouseDataBrowser/.

The mouse-to-human age-map can be found at: 

http://rubinlab.bgu.ac.il/mouseAPK/H2M/.

## Results

### The mouse-APK, knowledge mining and quality

As an extension to the APK, we developed the mouse-APK, aimed at capturing age and disease associations in mice as they are described in biomedical literature. The mouse-APK was developed using the same methodology we described for the human APK [12], but making minor adaptations in order to mine mouse-related abstracts. Starting with 501,648 abstracts associated with mice, 11,648 abstracts deemed relevant to age. For these, the mentioned age was extracted and textual snippets generated from these abstracts were mapped to terms from the Disease Ontology and a subset of UMLS concepts using a simple text-matching method (see Methods). Over 9000 instances generated by the text-mining pipeline, which mentioned both age and at least one phenotype, were used to populate the mouse-APK database.

**Figure 2:**
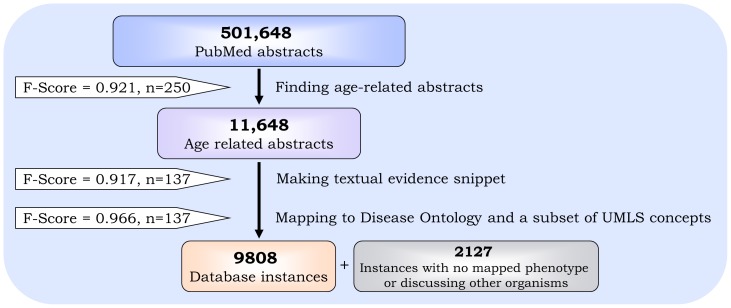
Knowledge mining pipeline. The process of mining the PubMed abstracts can be divided into three main steps: 1) Finding age-related abstracts, 2) generating a textual snippet which describes the most important information given in the abstract regarding the captured age and 3) mapping the text snippets to phenotypes from the DO and a subset of the UMLS. Instances for which no phenotypes were found, or were found to be linked to organisms other than mice were not used to populate the database.

The knowledge-mining process was evaluated by comparing the text mining results to those obtained by a human reader (Figure 2), similarly to the text mining evaluation we previously preformed [12]. The results suggest that the text mining process is accurate and that the resulting data is of good quality.

We provide users of the mouse-APK with a user-friendly query engine. Three types of queries can be performed with the search engine: 1) Search for evidence by age, 2) search for evidence by phenotype and 3) search for evidence by age and phenotype. The results can be refined by publication year, curation status, publication type etc. The query engine is freely available for use and can be found at: http://rubinlab.bgu.ac.il/mouseAPK/mouseDataBrowser/.

### Grouping diseases according to their age-related pattern

**Figure 3:**
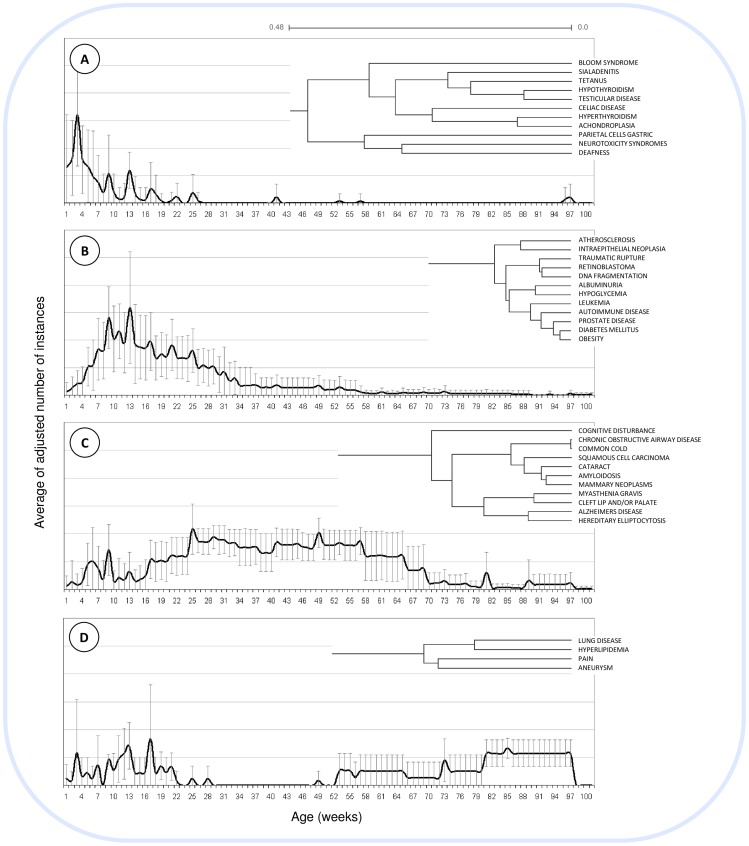
Hierarchical clustering results. Four examples of clusters are presented; for each, a phylogram and graphical representation of the average of corrected number of instances per age and disease is illustrated. Examples were chosen for their diversity in patterns.

To evaluate the ability of the knowledge stored in the mouse-APK database to correctly represent and summarize current knowledge, we conducted cluster analysis of the data, grouping together diseases that share similar age patterns. Many of the clusters thus generated demonstrate that these clusters mirror actual biomedical knowledge. Four representative clusters are described in detail (Figure 3). The first cluster (Figure 3a) involves several diseases (such as deafness, hypothyroidism, hyperthyroidism and celiac disease) which are investigated in younger mice. The second cluster (Figure 3b) demonstrates the clustering of diseases such as diabetes mellitus, obesity, albuminuria and leukemia which are investigated in slightly older mice than those represented in cluster 3a. Cluster 3c represents diseases which are more associated with older ages (such as cataracts, amyloidosis and Alzheimer's disease) and are investigated in mice in a much wider range of ages. The forth cluster (Figure 3d) represents diseases (pain, lung disease, hyperlipidemia and aneurysms) which, in mice, are associated with two separate age groups. The complete clustering results are provided in Figure S1.

### Mapping age between humans and mice

**Figure 4:**
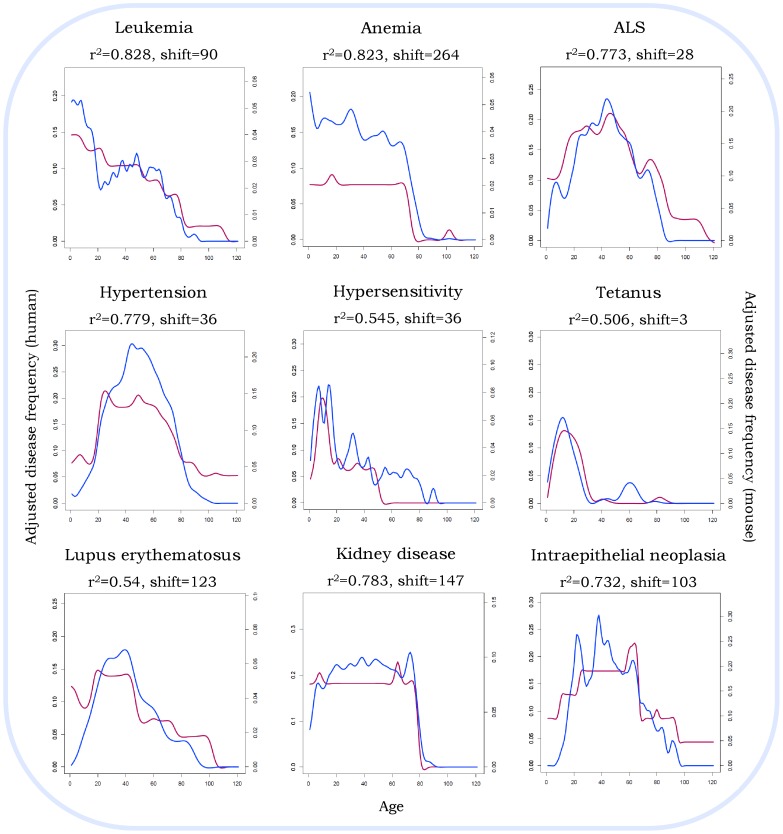
Correlating disease patterns between mice and humans. Nine of the 223 diseases that showed a correlation (r^2^> = 0.5) are illustrated here, chosen for best demonstrating similarity in patterns. The correlation was calculated based on adjusted disease frequencies in both species (based on data obtained from the corresponding databases, APK or mAPK). The correlation coefficient was calculated after considering every possible shift in patterns, choosing the shift that yielded the best correlation.. The patterns were splined for illustration purposes only.

Using the mouse-APK, we set out to map the most relevant age in mice for human diseases. This was achieved by comparing the age distribution pattern for 887 diseases in both species. These patterns were matched, allowing for age differences by shifting the patterns, and considering only distributions for which a high correlation (defined as r^2^>0.5, using Pearson correlation) was found. We successfully matched human-mouse age ranges for 223 diseases with this process. Nine such diseases (chosen for best demonstrating similarity in patterns) are depicted in Figure 4. It's interesting to note that the similarity does not stem from simple monotonous distributions; rather, each disease has a unique distribution over age, and the same distribution can be found, after adjusting for age-shifts, in mice as in humans.

### The mice-to-human age map

Once it was established that many diseases share similar patterns in age between mice and humans, especially when allowing some shift in age, it was possible to generate a mice-to-human age map. For each disease, the age shift that resulted in the highest correlation coefficient was used to generate a disease-specific age-to-age mapping between mice and humans. For example, for the disease Diabetes mellitus, the patterns are best correlated when there is a 99 point shift. The mouse age of 120 days would thus be mapped to the age 21 years in humans.

This age map is available to users at: http://rubinlab.bgu.ac.il/mouseAPK/H2M/.

**Figure 5:**
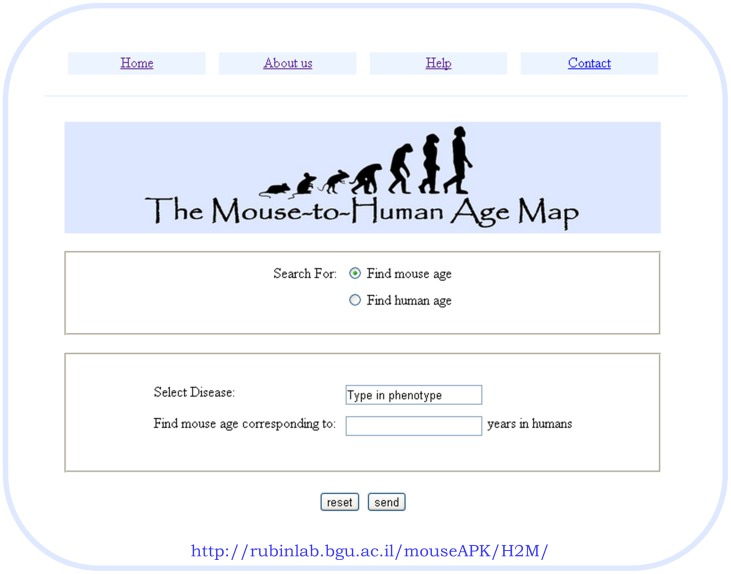
The Mouse-to-Human Age Map search tool. Users can search for a mouse age which correlates to a given human age or vice versa.

Using this tool, a user can enter the human age and disease they are interested in examining and receive the corresponding mouse age for that disease. Alternatively, the user can enter a mouse age and receive the corresponding age in humans for a given disease. Figure 5 illustrates a snapshot of the Mouse-to-Human Age Map search tool.

## Discussion

We present here the development of the mouse Age Phenome Knowledgebase and investigate possible approaches to bridging the gap between mouse models for disease and humans. The mouse-APK was populated with many instances mined from PubMed abstracts, using our own text mining pipeline. Considering the limitations of text mining techniques, such as incomplete or missing information, our pipeline was evaluated to have an overall good accuracy. We are also aware that our procedure may be missing some knowledge, such as that found in the full texts and not in the abstracts. However, the knowledgebase as it stands holds nearly 10,000 links between diseases and age, which make it sufficiently rich to be useful.

Using hierarchal clustering, we show that knowledge stored in the mouse-APK can be used to capture the current medical knowledge in at least two examples. In one example (Figure 3b), we find clustering of diseases that are associated with a wide range of ages. Several of these diseases, obesity, diabetes mellitus and hypoglycaemia, are known to be associated regardless of age [19],[20]. In the second example (Figure 3c), many of the so-called “age-related diseases” (such as Alzheimer's disease, cognitive disturbance, cataracts, amyloidosis and several types of cancer) are clustered together, mirroring current medical views [21]–[24].

There are obvious limitations to the approach we are presenting here. Perhaps most concerning is its sensitivity to research biases. Since the mouse-APK is populated with information extracted from published papers, the results of our analyses are influenced by the way diseases are investigated in mice and reported in the literature. However, capturing this knowledge can be very useful regardless to such limitations; since it includes the description of a large proportion of the experiments that were ever performed, it may be used to design novel experiments that would shed light on under-investigated age ranges. Another limitation of the current approach is the amalgamation of results obtained from several different murine models for the same disease, each investigated in different ages. While one model may exhibit symptoms at an early age, a second model for the same disease may only be useful at a later age. For example, the cluster depicted in Figure 3d illustrates diseases which are highly associated with two separate age groups, young and much older mice. This separation of the associations into two age groupings may be due to different models used to examine these diseases in different ages. In fact, in the case of hyperlipidemia, three database instances linking the disease with young mice (5 to 16 weeks of age) used a mouse model involving low-density lipoprotein (LDL) receptor-deficient mice [25]–[27] while two instances linking hyperlipidemia with older mice (27 to 64 weeks of age) involved ApoE deficient/knockout mice [28],[29]. While multiple models could explain, at least in part, the two age groupings seen in Figure 3d, for other diseases the effect of different models is not so clear. For example, in the case of ‘Pain’, many different models were associated with the phenotype and could not be clearly divided into two sets (models associated with young mice and models associated with older mice).

Nevertheless, our success in mapping many disease specific age distributions to humans and anecdotal examination of the results suggest that the lion's share of the data is only marginally affected by these distortions. If selection and reporting biases would have dominated the age of mice in experiments, we would have expected much more uniformity in mice ages. Yet we observe for many diseases a good fit in *the overall* distribution of ages (as seen in examples presented in Figure 4, and discussed in more details below). As for the possible interference from multiple models differing in typical age, this concern can be further alleviated in future versions of the mouse-APK, by more precisely capturing disease-model types and strains.

Our results demonstrate that many diseases share similar patterns in age between mice and humans. We note that the similarity extends beyond the major spike; for ALS, for example, two major spikes are observed for both species, and these are proportionally separated in time. It is also interesting to note that each disease required a different age-shift to maximize the similarity; in the examples brought here, this shift ranged from 3 to 264 days (in mice). This suggests that optimizing mouse models require the consideration of the optimal age of each disease separately. Furthermore, for many of diseases examined, significant correlation between the mouse and human age-related patterns could not be found even when allowing a shift in patterns. This could reflect a lack of age-equivalence between the two species; it possible that for some diseases, the distribution of relevant ages is too different from humans to be matched. These results strengthen the notion that there are many innate differences between humans and mouse models, although careful analysis is required to rule out other explanations (such as lack of data, higher error rates, large background/model induced variability etc.).

We therefore suggest that the biological gap between humans and mouse models, which limits our ability to translate findings between the species, may be bridged, at least to some extent, by more careful selection of the ages at which the mice are investigated. Our results thus indicate that using mice models can be fine-tune by allowing for more flexibility when comparing data patterns between the two species. Altogether, we believe our results indicated that it is possible, through knowledge mining, to propose ways to reduce the differences between mouse models and human patients.

There are numerous ways to improve the analysis. Perhaps the most obvious is to mine full papers rather than abstracts (both for humans and for mice). This requires sophisticated text mining techniques, as the complexity and shear volume of text are beyond the scope of the simple tools we have employed here. A second possible improvement is the search for more complex age mappings; we have used a very simple technique to detect similarities, namely shifting patterns relative to the other and searching for correlation. Using more sophisticated matching algorithms could reveal additional human-mouse disease associations which involve more restricted periods. Finally, including clinical or biological markers in the analysis, such as blood tests or gene expression profiles, could further help in detecting the fine age-patterns in each species, allowing more accurate and sensitive mapping to be achieved.

## Conclusions

To conclude, we present here the mouse Age Phenome Knowledgebase, which holds literature-derived information linking diseases, other phenotypes and ages. Using knowledge from the mouse-APK along with the equivalent human knowledgebase, we describe a novel approach aimed at diminishing the contribution of inadequate age choice to differences between mouse models and the target human patients. To the best of our knowledge, this is the first systematic attempt to bridge age differences between murine models and humans and may lead to better experimental design and outcome.

## Supporting Information

Figure S1 Hierarchical clustering results. The complete results of hierarchical clustering of diseases based on age-related disease patterns.
(JPG)

File S1 The text mining script (Miner_mouse.pl) implemented in Perl. This script was used to mine PubMed abstracts for age associations.
(ZIP)

File S2 The text mining script (Mapper_mouse.pl) implemented in Perl. This script was used to map age-related abstracts (found by Miner_mouse.pl) to terms from the Disease Ontology, a subset of the UMLS and a list of mouse strains.
(ZIP)

File S3 Disease Ontology parsing script implemented in Perl. This script generates the Disease Ontology name/synonym file used by Mapper_mouse.pl.
(ZIP)

File S4 Age, relation and strain terms. This file contains lists of age and relation terms used as regular expressions by the Miner_mouse.pl script and a list of mouse strains used by the Mapper_mouse.pl script.
(ZIP)

File S5 Text mining evaluation results. This file contains the complete evaluation results of the text mining pipeline.
(XLS)

File S6 Mouse data matrix. A quantitative age-disease matrix generated by obtaining, for each disease mapped in the database, a count of the number of instances mapped to that disease per age. Evidence linked to inferred age ranges was excluded.
(TXT)

File S7 Correlation comparison results. A comparison of one-day resolution and one-week resolution in disease patterns correlations between mice and humans.
(XLS)
