# Supplementary figures and images for: The Mouse Age Phenome Knowledgebase and Disease-Specific Inter-Species Age Mapping
Source: PLoS One. 2013 Dec 3;8(12):e81114. doi: 10.1371/journal.pone.0081114 (PMC3849212; doi:10.1371/journal.pone.0081114)

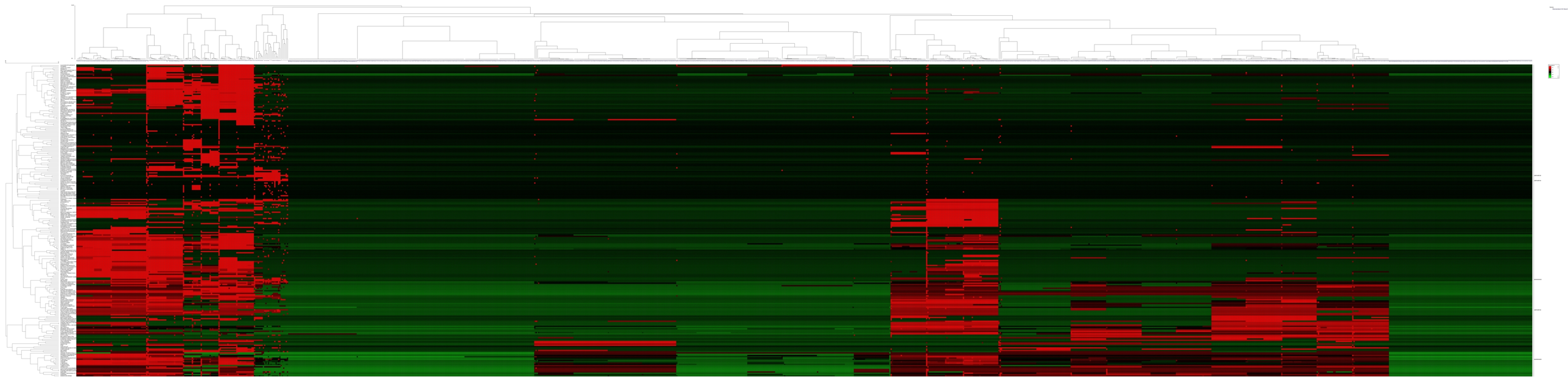

Supplement: Figure S1 — Hierarchical clustering results. The complete results of hierarchical clustering of diseases based on age-related disease patterns. (JPG) [file pone.0081114.s001.jpg]
